# A Fully-Differential Switched-Capacitor Dual-Slope Capacitance-To-Digital Converter (CDC) for a Capacitive Pressure Sensor

**DOI:** 10.3390/s19173673

**Published:** 2019-08-23

**Authors:** Christopher Rogi, Cesare Buffa, Niccolo De Milleri, Richard Gaggl, Enrique Prefasi

**Affiliations:** 1 Infineon Technologies Austria AG, RF & Sensors, Siemensstr. 2, 9500 Villach, Austria; 2 Electronic Technology Department, Carlos III University of Madrid, 28911 Madrid, Spain

**Keywords:** MEMS, SPICE model, electro-mechanical coupled simulation, pressure sensor, CDC, Capacitance-to-Digital Converter, Dual-Slope, noise-shaping, auto-zero, switched-capacitor

## Abstract

This article focuses on a proposed Switched-Capacitor Dual-Slope based CDC. Special attention is paid to the measurement setup using a real pressure sensor. Performance scaling potential as well as dead zones are pointed out and discussed. In depth knowledge of the physical sensor behavior is key to design an optimal readout circuit. While this is true for high-end applications, low-performance IoT (Internet of Things) sensors aim at moderate resolution with very low power consumption. This article also provides insights into basic MEMS (Micro-Electro-Mechanical-System) physics. Based on that, an ambient air pressure sensor model for SPICE (Simulation-Program-with-Integrated-Circuit-Emphasis) circuit simulators is presented. The converter concept was proven on silicon in a 0.13 μm process using both a real pressure sensor and an on-chip dummy MEMS bridge. A 3.2-ms measurement results in 13-bit resolution while consuming 35 μA from a 1.5-V supply occupying 0.148 mm^2^. A state-of-the-art comparison identifies potential room for improvements towards hybrid solutions, which is proposed in subsequent publications already.

## 1. Introduction

Pressure sensors that convert gases or liquid pressure into an electrical signal are widely used in several fields, such as automotive, consumer, medical and industrial. Thus, they play a leading position in the sensors market and become a more and more important component for Internet-of-Things (IoT) applications. Among all available options, Micro-Electro-Mechanical-Systems (MEMS) technology is the main choice for pressure sensors for low-pressure and small size applications. Only for pressures higher than ≈1000 bar, thin-film technology becomes attractive. The success of MEMS technology for pressure and many other sensors is the possibility to combine μ-size mechanical sensing elements and adequate resolutions with extremely low-power consumption and low fabrication costs with standard photographic processes [1].

Automotive applications (TPMS (Tire-Pressure-Monitoring-System), side airbags, particles filters, etc.) have always been leading the pressure sensor market. It is still the largest in terms of sold parts and revenues. Nevertheless, the second most dynamic market position belongs to consumer applications, where pressure sensors become more and more popular thanks to emerging realities and goods, such as drones, wearables, indoor navigation, augmented reality, etc. For the consumer market, pressure sensors are typically available in combination units together with accelerometers and gyroscopes, or as monolithic integration. The latter solution is gaining popularity and also reaches very small packages. As a consequence, the power consumption and cost can also be reduced to meet the typical consumer market specifications. Mainly two different types of MEMS pressure sensors are available as stable technology, namely piezo-resistive and capacitive. The first category is more suitable to implement differential measurements, while the latter is well suited for ultra-low power sensing with good temperature compensation and small die size [1].

Even though the MEMS technology for pressure sensors is quite mature, naturally, there is always a need for lower-power and higher-resolution architectures to read-out capacitive sensors. While mobile pressure sensing applications (e.g., wearable devices, drones, etc.) need to maximize battery run time, stationary IoT (Internet of Things) applications may need high resolution. Additionally, the majority of intelligent sensing systems is digital with a shared computing unit to interpolate data from different sensors. In this scenario, Capacitance-to-Digital Converters (CDCs) represent the ultimate state of the art for capacitive sensing interfaces, showing a high resolution vs. conversion energy ratio. They also provide a direct conversion of the physical quantity into a digital word.

Various CDC architectures have been reported in the literature. This includes approaches based on Successive-Approximation-Register (SAR) [2], Period-Modulation (PM) [3], Pulse-Width-Modulation (PWM) [4], Delay-Chain discharge (DC) [5], Delta-Sigma (ΔΣ) modulators [6] and Dual-Slope (DS) [7] Analog-to-Digital Converters (ADCs). While SAR ADCs offer fast conversion speed. the matching requirements often limit their performance. Approaches based on PM/PWM offer an intrinsic semi-digital nature as quantization is performed by a digital counter. However, oscillator based concepts usually need dynamic calibration due to stronger dependence on process and temperature. On the other hand, ΔΣ modulators can achieve high converter accuracy due to oversampling and noise-shaping. However, often a Charge-to-Voltage (C/V) stage is needed to interface the sensor. A direct Switched-Capacitor (SC) sensor readout is possible at the cost of increased sensor power consumption. Finally, Dual-Slope based CDCs can provide a simple and inherently robust topology. Nevertheless, without further improvements those converters usually lack behind in conversion speed.

In this article, the physical basics of MEMS sensors are discussed first. Based on that, a SPICE model approach of an ambient air pressure sensor is presented. Next, a noise-shaping direct CDC based on a complete SC Dual-Slope converter is proposed to read out a particular pressure sensor [8]. Due to a switched-capacitor sensor readout, no additional interface stage is needed. A single OTA (Operational Transconductance Amplifier) performs both sensor readout and digital conversion. Auto-zeroing reduces OTA matching and low-frequency noise requirements. Quantization noise-shaping within the Dual-Slope operation decreases the measurement time [9]. A single-bit capacitive DAC is used during digitization. To generate the multi-bit output, only single-bit circuitry and a counter is used. Utilizing the same reference voltage in both Dual-Slope phases the CDC robustness is improved. A digital averaging filter calculates the final digital result.

Prototypes of this CDC are implemented in a 0.13 μm standard CMOS process. After elaborating on the measurement setup, the main measurement results are presented. This involves both a real pressure sensor MEMS and a on-chip dummy bridge for CDC testing.

## 2. Capacitive MEMS Physics

A typical single-ended capacitive pressure sensor can be modeled, from an electrical point of view, as a parallel-plates capacitor: a “small” displacement (with respect to the distance to the anchored electrode) of the moving mass gives a capacitance variation. Therefore, capacitive variations measurement is at the base of this readout technique.

**Figure 1:**
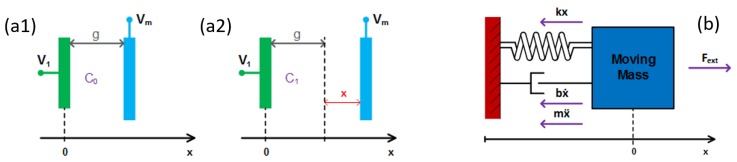
MEMS modeled as a variable single-ended parallel-plates capacitor: (**a1**) at rest position; (**a2**) at a displacement *x* with respect to rest position; and (**b**) lumped parameters model of a 1-DOF spring–mass–damper system with the balance of forces acting on the micro system.

At rest position, neglecting mechanical offset and fringing fields (which is a reasonable approximation when plates length and height are much longer compared to the distance between two electrodes), the capacitance formed by a movable plate with a stator is given by

(1) $$\begin{array}{c} C_{0}=\frac{\varepsilon_{0}\cdot A_{C}}{g}\;, \end{array}$$

where *g* is the distance between two electrodes (gap) at rest, $A_{C}$ is the overall sensing electrodes area and $\varepsilon_{0}$ is the vacuum dielectric constant (Figure 1a1). In the case of a displacement *x*, the sensor capacitance becomes

(2) $$\begin{array}{cc} C_{1}\left(x\right) & =\varepsilon_{0}\cdot \frac{A_{C}}{\left(g+x\right)}\;. \end{array}$$

Figure 1a2 shows the displacement of the moving mass in presence of an external force and its effect on the capacitance variation. Because of opposite charges on two plates forming a capacitor, there is a force of attraction between plates, which is commonly neglected in fixed-plates electrical capacitors. This charge is always present whenever the capacitor is charged and, in the case of at least one movable plate, the inclusion of this mechanical force becomes essential.

A MEMS can be modeled as a lumped parameter spring–mass–damper system, as shown in Figure 1b: a mass is connected via a spring to a fixed support, being pulled by an external force $F_{ext}$. A dashpot is used to represent a mechanical damping element. Considering that all these three elements share the same displacement *x*, and applying Newton’s second law of motion, the classical equation of motion can be derived [10].

**Figure 2:**
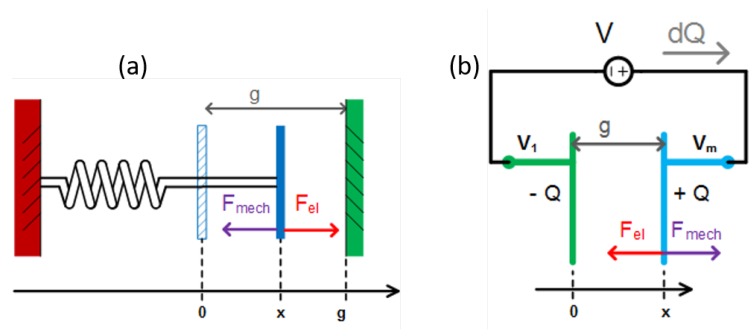
(**a**) Equilibrium on the moving mass considering an electrostatic force; and (**b**) the corresponding charge balance with a voltage biasing scheme.

Additionally, when a MEMS capacitor is voltage-biased, an electrostatic force between the two electrodes raises, which depends on the distance between the electrodes themselves. This is illustrated in Figure 2 for both an (a) mechanical and (b) an electrical point of view. For a limit case where electrodes distance tends to 0, the electrostatic force diverges to very high values:

(3) $$\begin{array}{c} F_{elecstat}=\frac{1}{2}\frac{\delta C}{\delta x}\cdot {\left(V_{1}-V_{m}\right)}^{2}=\frac{1}{2}\cdot \varepsilon_{0}\cdot A_{C}\cdot \frac{{\left(V_{1}-V_{m}\right)}^{2}}{{\left(g+x\right)}^{2}}\;. \end{array}$$

Considering this force, the new equilibrium of the moving mass is determined by a refined equation of motion following

(4) $$\begin{array}{c} m\ddot{x}+b\dot{x}+kx+F_{elecstat}=F_{ext}\;. \end{array}$$

Depending on the operating conditions and values of parameters, balance of forces can be dominated by a specific component and Equation (4) can have different solutions, which are either stable or unstable. In the case of voltage controlled parallel-plate capacitors, an important behavior called *pull-in* might happen: at some critical voltage the system becomes unstable and the gap collapses to zero. Following a stability analysis of the equilibrium between elastic force and electrostatic force, it can be shown that pull-in occurs at [11]

(5) $$\begin{array}{c} x_{pi}=\frac{g}{3}. \end{array}$$

With this value of displacement, the equilibrium voltage is

(6) $$\begin{array}{c} V_{pi}=\sqrt{\frac{8}{27}\cdot \frac{k\cdot g^{3}}{\varepsilon_{0}\cdot A_{C}}}\;. \end{array}$$

Equation (6) provides the pull-in voltage for a single-ended parallel-plates capacitor [12].

## 3. MEMS Modeling Approach

Compact modeling of MEMS sensors aims at low computational complexity and good accuracy. It is about describing the transducer’s physical and electrical behavior in the frequency range of interest. The design of the sensor readout circuit is optimized using an embedded sensor model in traditional electrical simulators. In this case, the full signal chain is considered. The model includes basic physics of the transducer while the readout circuit operates with equivalent electrical quantities. Additionally, the integrity and complexity of such sensor models can be tailored depending on the needs of each phase of a development project.

**Figure 3:**
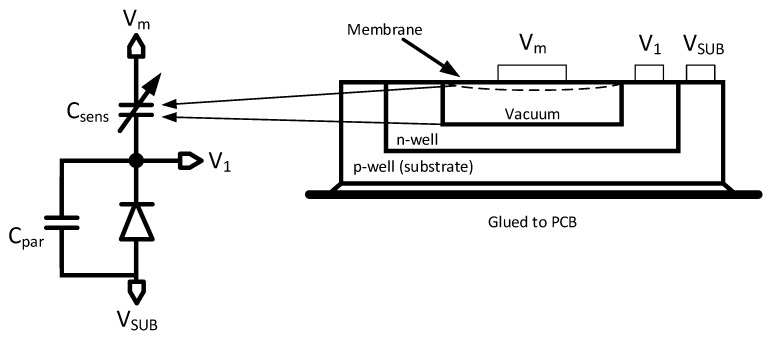
Schematic equivalent and physical MEMS sensor view.

**Figure 4:**
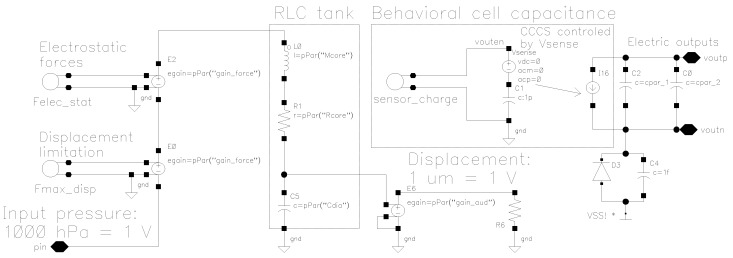
SPICE model of one single MEMS pressure sensor cell.

In this article, arrays of single MEMS pressure sensors cells are of interest. In the following, the model of a single sensor cell is derived. The simplest and most efficient way to describe the basic functionality of systems within a traditional electric simulator is by exploiting second-order systems. Therefore, the use of electro-mechanical analogies is favored. In Figure 3, an example of a basic model of a single pressure sensor cell can be seen. The sensor cross section to the right shows the potential movement of the membrane due to pressure or bias voltage variations. This leads to an equivalent capacitor $C_{sens}$, which changes the capacitance value accordingly. Consequently, the model contains the basic analytical capacitance and electrostatic force functions against membrane displacement. The schematic of an equivalent spice model is shown in Figure 4.

An important input parameter of the model is the externally applied pressure. In this model, 1 bar (=1000 hPa) pressure is equivalent to 1 V at the spice model pin. Based on the input pressure and the applied bias voltage the electrical model reacts by calculating the charge stored in the MEMS cell and deriving the current flowing through the device.

The mechanical dynamic domain is modeled with the RLC (Resistive-Inductive-Capacitive) tank in which the mass of the membrane is represented by the inductive element, the compliance by the capacitance, and the damping by the resistive element. The electro-mechanical forces and the limits of the membranes displacement are included through mathematical functions to enhance accuracy and aid simulator convergence, respectively. The electric interface of the system can be seen as a behavioral active MEMS capacitor that shows complex impedance depending on the frequency of excitation. Further mathematical details follow below.

Equation (2) shows the MEMS-cell capacitance as a function of membrane displacement (*x*) described with the analytical parallel plate capacitance. More advanced versions are usually obtained through finite element analysis of the structure or direct measurements and can be similarly modeled with higher order polynomial approximations as a function of membrane displacement. The model of the electrostatic forces in Figure 4 are obtained as the derivative of the potential energy stored in the MEMS capacitance with respect to the membrane displacement. In the analytical case, this results in Equation (3).

The membrane displacement limitation block in Figure 4 counteracts the external forces applied to the membrane (both acoustic and electrostatic) when the membrane has reached the maximum allowed displacement. Such a block needs to be optimized for solver convergence as it puts a hard limit to a continuous signal. This might result in discontinuities and numerical issues. To mitigate this issues, a possibility is to use analog switch relations that rely on a continuous mathematical functions such as the hyperbolic tangent. The hyperbolic tangent allows trimming the sharpness and the accuracy of the transition from one mode to the other. As a result,

(7) $$F_{maxdisp}=-\frac{1+tanh\left(\frac{V\left(gap\right)-f_{c}\cdot V_{trig}}{V_{trig}}\right)}{2}\cdot \left(F_{elecstat}+F_{pressure}\right)$$

is applied as a limiting function in Figure 4, where $F_{pressure}$ represents the externally applied pressure equivalent to $F_{ext}$ in Equation (4). The switch transition error $err_{@tr}$ is tuned via the $f_{c}$ parameter

(8) $$err_{@tr}=\frac{1+tanh\left(-f_{c}\right)}{2}.$$

Furthermore, the transition time $t_{rise}$ is used to parametrize $V_{trig}$ through

(9) $$V_{trig}=\frac{t_{rise}}{tanh^{-1}\left(1-err_{@tr}\right)+f_{c}}.$$

Finally, the current-controlled current source in Figure 4 represents the sensor model interface. The current through $V_{sense}$ is modulated by the different forces within this sensor cell model. Depending on accuracy requirements, such a model can be further extended to include temperature or stress effects.

**Figure 5:**
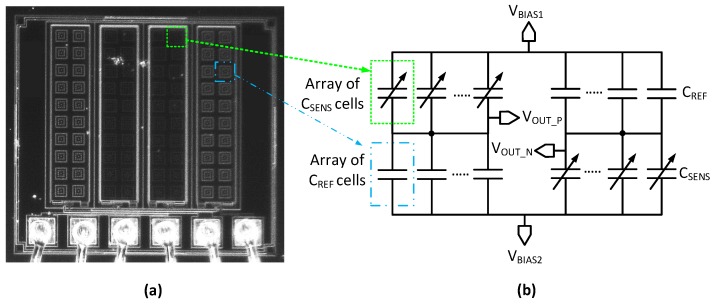
MEMS die photograph (**a**); and equivalent model schematic based on sensor and reference cell arrays (**b**) without parasitic capacitance.

Thus far, a model of a single MEMS sensor cell is described. A complete sensor die usually contains several sensor cells, such as the one used in this work in Figure 5a. In this case, it is convenient to simply combine an adequate amount of sensor cell model instances to form a versatile model of the full sensor-bridge. Figure 5b shows a fully-differential sensor bridge model comprised of multiple sensor and reference cells. Each sensor cell is modeled with an independent model instance. Similarly, a model for the reference cells can be designed and implemented.

One advantage of a bridge configuration is that the difference between the sensor and reference is measured, rather than the absolute sizes. The presented pressure sensor covers an application range of

(10) $$0.3<p_{range}<1.2\;\left[\mathrm{bar}\right].$$

**Figure 6:**
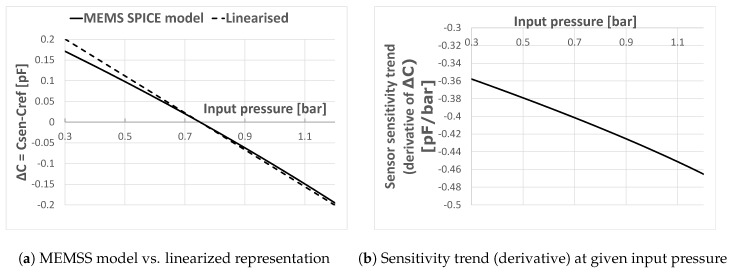
ΔC reading of sensor model (**a**); and sensitivity (derivative) (**b**).

For optimum swing, the sensor reading must be centered, as discussed in Section 4. The presented (centered) spice model simulated over the full pressure range is shown in Figure 6a. It represents the differential difference between the sensor and reference capacitor. Note that the presented spice model also covers sensor non-linearity. This can be further observed in Figure 6b, where the derivative of the ΔC reading is plotted. This non-linearity is due to varying membrane stiffness rather than parasitic effects.

In [8], a Capacitance-to-Digital Converter (CDC) is proposed. In the next sections, this CDC reading the real MEMS sensor is elaborated. It is shown below that certain circuit parameters heavily depend on the sensor sensitivity. To design proper CDC programmability, a linearized sensitivity estimation is sufficient, as shown in Figure 6a.

## 4. Sensitivity Linearization of the Real Pressure Sensor MEMS Full-Bridge for the Design Process

For optimum performance, the CDC full scale must be adjusted to a given sensor sensitivity. Dependent on the MEMS production process spread, the membrane sensitivity to external air pressure varies. Equations (15) and (16) show that some circuit parameters are directly related to the sensor sensitivity. To provide a suitable capacitor array for $C_{F}$ and $C_{DAC}$, the available sensor sensitivity must be anticipated. This assessment was done by the pressure sensor MEMS development team at Infineon. Table 1 provides details on the pressure sensor MEMS. It states both the absolute capacitor sizes and their variation over a given ambient air pressure range from best case to worst case. It can be seen that, at the highest sensitivity, the difference between $C_{sen}$ and $C_{ref}$ is around 782 fF over the application pressure range. On the other hand, low performing MEMS sensitivity is as low as 235 fF.

**Figure 7:**
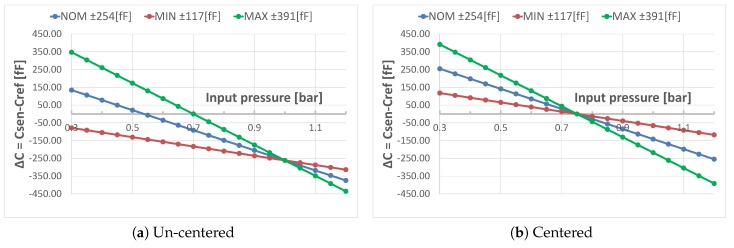
Single-ended un-centered (**a**) and centered (**b**) linearized equivalent pressure sensor ΔC reading over application input range.

A linearization of the data in Table 1 is shown in Figure 7a. A different slope indicates a different sensitivity of the sensor over the input pressure. An additional offset compensation is required in order to center the ΔC reading of the CDC. This adjusts the CDC full scale to an optimum of $\pm C_{FS}$. Since the input pressure range is from 0.3 to 1.2 bar the center is at (0.3+1.2)/2=0.75 bar.

A centered sensitivity linearization is shown in Figure 7b. The effect of different sensitivities is pointed out even more. Note that this also represents the ideal CDC sensor reading. While a zero reading represents 0.75 bar, ± the digital full scale reading is equivalent to $\pm C_{FS}$.

## 5. A Switched-Capacitor Noise-Shaping Dual-Slope direct CDC

**Figure 8:**
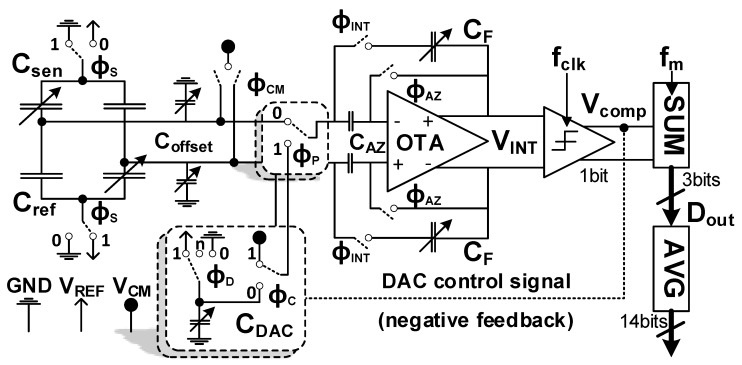
Implemented fully differential Switched-Capacitor direct CDC. Note: DAC and $\phi_{P}$ have single-ended representation for more simplicity.

To read out the pressure sensor, a dedicated ASIC (Application Specific Integrated Circuit) must be developed. In this case, a digital sensor representation is generated based on a capacitive sensor reading. This functionality defines a CDC (Capacitance to Digital Converter). The converter ASIC is connected to the pressure sensor via bond wires. The full CDC topology is shown in Figure 8. The discussed differential capacitor sensor bridge in Section 4 is directly connected to the CDC. The bridge consists of two sensing and two reference capacitors. Due to the bridge configuration, the absolute value of the capacitors is canceled and the differential value is measured. Note that there is no dedicated interface stage needed due to a direct switched-capacitor readout, similar to George and Kumar [13]. The OTA is used within a SC integrator. Potential OTA offset and flicker-noise is reduced by auto-zeroing. The trimmable on-chip capacitors $C_{offset}$ are used to optimize digital full scale swing of the CDC.

The dual-slope based conversion cycle consists of two phases: Phase I integrates the sensor charge difference, which represents the signal of interest in this system. Phase II in the dual-slope conversion evaluates the integrated sensor charge using a differential SC DAC. One multi-bit conversion cycle takes

(11) $$T_{m}=\frac{1}{f_{m}}=\left(N+M\right)\cdot T_{clk}=\left(N+M\right)\cdot \frac{1}{f_{clk}},$$

where *N* and *M* represent the number of clock cycles during Phase I and Phase II, respectively. Multi-bit output is generated by summing *M* single-bit values of a clocked comparator. Consequently, the multi-bit signal runs at a lower rate $f_{m}$, while the single-bit system runs at $f_{clk}$. For first measurements, the multi-bit signal is averaged by a off-chip digital averaging filter to calculate the final result.

**Figure 9:**
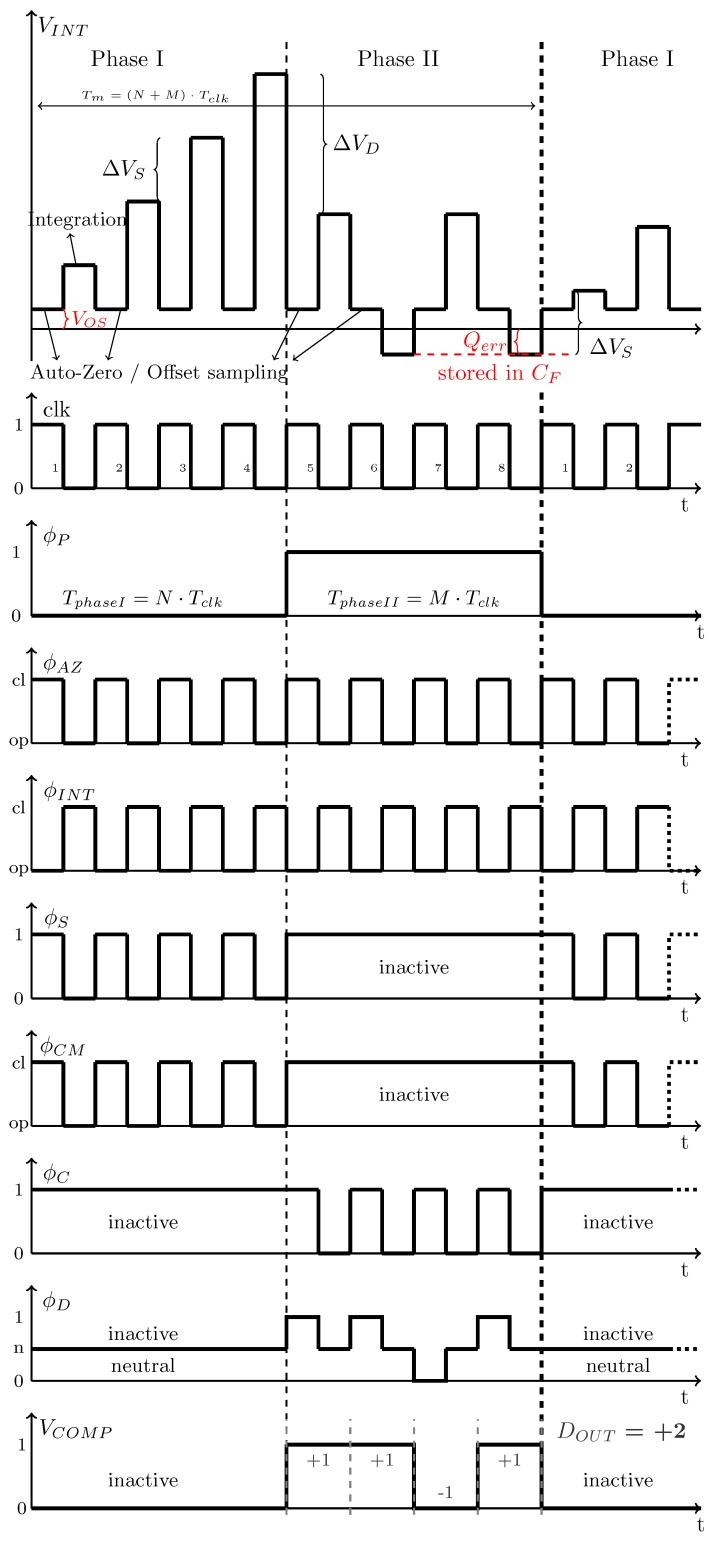
Implemented timing diagram and data evaluation of N=M=4. Note: Non-overlapping signals are not shown and infinite OTA settling speed is assumed for more simplicity.

In Figure 8, the switches $\phi_{P}$ are used to change between the two phases. Note that both phases are based on a switched-capacitor approach. Therefore, it is possible to insert OTA auto-zeroing within both phases. A more detailed description of the CDC operation is given in the following. Figure 9 shows the corresponding timing diagram.

### 5.1. Phase I: Switched-Capacitor (SC) Sensor Readout

Each multi-bit conversion cycle $T_{m}$ starts by pre-charging the four sensor bridge capacitors to $V_{REF}-V_{CM}$ and $GND-V_{CM}$, respectively (via $\phi_{S}$ and $\phi_{CM}$ in Figure 8) [13]. At the same time, the OTA is auto-zeroed using unity gain feedback ($\phi_{AZ}$). The switches $\phi_{P}$ are connected to position 0 while the switches $\phi_{CM}$ are closed. The OTA offset is sampled on the dedicated offset sampling capacitors $C_{AZ}$. Next, at the negative clock edge, the OTA is switched into integration mode via switches $\phi_{INT}$. In addition, the sensor switches $\phi_{S}$ toggle their position and switches $\phi_{CM}$ and $\phi_{AZ}$ open. Hence, the charge difference of the sensor bridge capacitors ($C_{sen}-C_{ref}$) is integrated onto the feedback capacitors $C_{F}$. The offset sampling capacitors $C_{AZ}$ are now in series with the bridge and the OTA offset is ideally canceled. Considering the finite OTA DC gain (*A*) the differential integrator output changes by

(12) $$\Delta V_{S}=\frac{2\cdot \left(C_{sen}-C_{ref}\right)\cdot V_{REF}}{C_{F}+\frac{C_{sen}}{A}+\frac{C_{ref}}{A}+\frac{C_{F}}{A}}$$

each clock cycle during Phase I (see Figure 9).

This procedure is repeated for *N* clock cycles within Phase I. The integrator output voltages changes by $V_{INT_{PhaseI}}=\Delta V_{S}\cdot N$ during Phase I. This assumes that the bridge capacitors do not change meanwhile. Note that $V_{INT_{PhaseI}}$ is directly proportional to the sensor bridge capacitor difference. In this implementation, N=4 has been selected.

### 5.2. Phase II: Digitization of $V_{INT}{|}_{\phi I}$ via a SC DAC

Phase II is used to digitize the measured bridge signal obtained during Phase I. The switching behavior is similar to Phase I. However, instead of the sensor bridge a single-bit capacitive DAC ($C_{DAC}$) is connected to the integrator. Therefore, the switches $\phi_{P}$ are closed at position 1. The same reference voltages $V_{REF}$ and GND as for the bridge during Phase I are used. The comparator is evaluated at each positive clock edge during Phase II. Depending on the comparator output $V_{COMP}$ the DAC capacitors $C_{DAC}$ are pre-charged to $V_{REF}$ or discharged to GND to form a negative feedback loop via switches $\phi_{D}$. Meanwhile, the OTA is again auto-zeroed. At the negative clock edge, the DAC charge is integrated on the integrator capacitors $C_{F}$. Depending on the comparator decision the differential integrator output voltage changes by

(13) $$\Delta V_{D}=\pm \frac{2\cdot C_{DAC}\cdot \left(V_{REF}-V_{CM}\right)}{C_{F}+\frac{C_{DAC}}{A}+\frac{C_{F}}{A}}$$

each clock cycle during Phase II (Figure 9). As a difference to a conventional Dual-Slope approach, the operation of the feedback DAC is not stopped after the first comparator sign change detection. Instead it keeps toggling around $V_{INT}=0$ using the DAC until the end of Phase II (intended oscillation). Furthermore, the integrator is not reset at the end of Phase II. Thus, the quantization error of each conversion remains stored in $C_{F}$. It has been shown in [14,15] that this method reveals first-order noise-shaping. Note that in [14] a continuous-time approach has been used.

During Phase II, the multi-bit digital data is obtained by using a counter. At each positive clock edge in Phase II the output of the comparator delivers either +1 or −1. Thus, after Phase II, *M* single-bit values are summed up to a signed $log_{2}\left(M\right)$ bit value. To obtain a 3-bit output signal, M=4 has been selected (2-bit + sign). It can be shown that the transfer function follows a mid-tread quantizer. One multi-bit conversion period takes $T_{m}=\left(N+M\right)\cdot T_{clk}$ (Equation (11)). Acquiring *K* multi-bit samples results in a total measurement time of

(14) $$T_{m_{total}}=K\cdot T_{m}.$$

A digital filter off-chip averages the *K* counter samples to get the final higher resolution CDC result. It represents a single digital value with high absolute accuracy based on an average value of many multi-bit conversions.

### 5.3. INtegrator Output Voltage Scaling via $C_{F}$ and $C_{DAC}$

For proper operation the OTA output stage devices must always stay in saturation region. Similar to scaling a ΔΣ integrator state variable the output voltage can be controlled by dimensioning $C_{F}$ and $C_{DAC}$ properly. For a desired maximum differential output swing $V_{swing}$ the integration capacitor $C_{F}$ is set according to

(15) $$C_{F}=2\cdot V_{REF}\cdot dC_{FS}\cdot \frac{\left(\frac{N}{M}+N\right)}{V_{swing}},$$

where $dC_{FS}$ represents the sensors sensitivity. Note that Equation (15) already considers the maximum remaining quantization error after Phase I. Consequently, the feedback DAC must be dimensioned using

(16) $$C_{DAC}=2\cdot dC_{FS}\cdot \frac{N}{M}.$$

Note that Equation (16) implicitly assumes $V_{REF}$ to be the DAC reference voltage. Table 2 summarizes the main parameter dimensions used. The real sensor sensitivity has to be derived via a CDC center calibration routine.

### 5.4. Circuit Design

In Figure 8, a Dual-Slope based CDC overview scheme is shown. There are only two active components involved, namely the OTA within a switched-capacitor integrator and a single-bit comparator. All capacitors (except $C_{sen}$ and $C_{ref}$) are implemented as metal VPP (Vertical Parallel Plate) capacitors using four metal layers. For testing the switched-capacitor Dual-Slope CDC approach, a traditional folded cascode OTA is implemented. This topology is preferred compared to, e.g., a telescopic OTA due to increased output swing while sill maintaining sufficient gain. Similar to a ΔΣ modulator, the output swing of the integrator is controlled mainly by proper dimensioning of the feedback capacitor $C_{F}$ (state scaling). Increased OTA swing capabilities help to minimize the dominant capacitor area requirements, as indicated in Equation (15). The effect of flicker-noise (1/f) is reduced due to OTA auto-zeroing. Note that in Figure 8, a dedicated capacitor $C_{AZ}$ is used for that purpose. It can be shown that, instead, the low-frequency components could also be sampled onto the sensor capacitors. This is beneficial, since, due to the switching nature, $C_{AZ}$ introduces significant kT/C noise.

A two-stage clocked comparator is used to convert the integrator output to a single-bit PWM waveform. It is directly used to control the feedback switches and internally buffered for external readout. It is important to point out that both the counter (summation) and averaging filter are implemented externally using MATLAB^®^.

## 6. Measurement Setup Details

In the following, details about the physical implementation and the measurement setup are given. Various package and chip photos provide further insights, followed by an overview of used auxiliary circuitry and measurement hardware. For the CDC, a 0.13 μm standard CMOS process is used.

**Figure 10:**
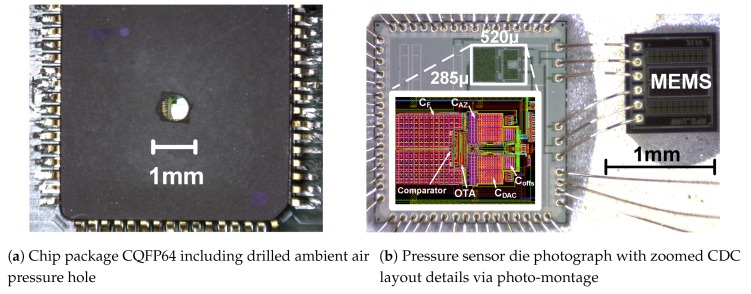
Chip photos: Package with air pressure hole (**a**); and opened package view including layout (**b**).

Figure 10b shows a photo-montage of the ASIC (Application-Specific-Integrated-Circuit) layout and the packaged composition of ASIC and MEMS sensor. The capacitor arrays $C_{F}$ and $C_{DAC}$ are designed to match to the predicted full scale according to Table 1 using Equations (15) and (16), respectively. In fact, it can be seen that the passive components require the majority of the CDC area. A narrower sensor sensitivity spread is therefore beneficial to minimize the ASIC area. Compared to a folded cascode OTA, a two-stage comparator as active components requires a fraction of the area.

**Figure 11:**
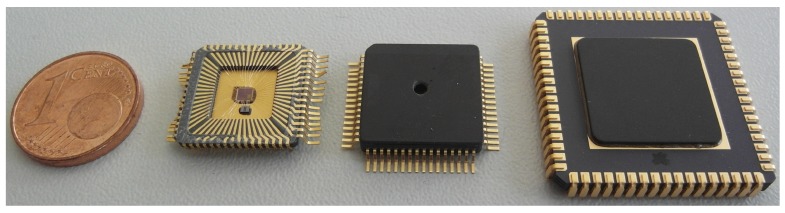
Chip size comparison. From left to right: one euro cent coin, real pressure sensor open package, real pressure sensor closed package, and on-chip dummy bridge ASIC packaged.

The two silicon dies are packaged in a CQFP64 package. Naturally, the sensor must be exposed to the ambient air pressure. Therefore, a small hole is drilled into the top lid of the package (Figure 10a). A closer look through the hole reveals the bonded ASIC beneath the lid. To better grasp the absolute size, a comparison is given in Figure 11. While two real pressure sensors are shown in the middle, the far right package contains an ASIC variant with an on-chip programmable dummy bridge for testing.

**Figure 12:**
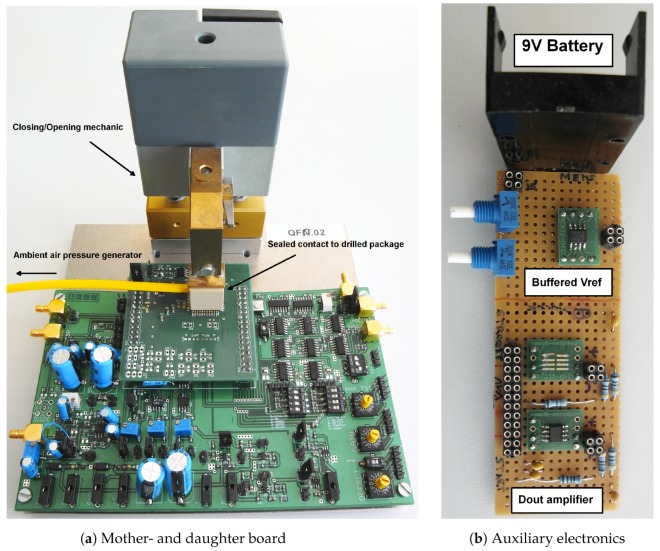
Motherboard attached to a daughter board with a real pressure sensor test chip docked to the ambient air pressure generator via pneumatic mechanics (**a**); an external reference generator (1.5 V from a 9 V block battery) buffer AD8034 and auxiliary digital output amplifier buffer ADN466 (**b**).

To connect the test chips, special discrete hardware is used. Both a mother- and daughter board are used to connect the test chip to auxiliary circuitry (i.e., test-bits programming, filters, supply generators, readout amplifiers, etc). More importantly, a hardware connection to a special pressure chamber is required to expose the sensor to the required pressure at full scale. Figure 12a shows the mother- and daughter board with the real pressure sensor on top. Special mechanics connect the sealed pressure chamber to a pressure generator through a pneumatic system.

Section 5 describes the use of a reference voltage $V_{REF}$. In all presented test chips, this reference voltage can be either generated on-chip (via an LDO) or applied externally. While the performance was similar, the external voltage application enabled the convenient current consumption measurement possibility. Figure 12b shows the external auxiliary circuitry. The reference voltage is derived from a 9-V block battery (low noise) where trimmable potentiometers gave further reference voltage tuning comfort. Furthermore, another buffer circuit helps to amplify the digital single-bit output of the CDC comparator.

### Used Measurement Hardware Utilities

The measurements were performed in the Laboratory at Infineon Technologies Austria AG in Villach. Various high quality equipment was used to perform steady measurements:

1. Ambient air pressure control unit: Druck Pace 5000
2. Oscilloscope: Tektronix DPO 5034
3. Clock generator: Tektronix AFG 310.2
4. Digital data capture: byte paradigm GP-24132
5. Voltage supply: Agilent E3631A with additional low-pass filters
6. Digital multimeters: Keithley DMM7510

The digital data capture unit represents the interface to a PC. For the digital post processing, MATLAB^®^ was used.

## 7. Main Measurement Results

In the following, the CDC ASIC is evaluated. Measurements of both an on-chip dummy sensor and a real pressure sensor bridge are presented. For all measurements, the single-bit comparator output is sensed and processed off-chip. Multi-bit conversion and the dedicated averaging filter are implemented via MATLAB^®^.

As pointed out in Section 5, several multi-bit conversions are averaged to derive a final higher resolution digital sensor value. This means that the signal information of the multi-bit data stream is at DC. Usually, the performance is evaluated using the Signal-to-Noise Ratio (SNR). To determine this, both the signal and noise power within a certain bandwidth is required. For DC signals, this fact often leads to discussions and misunderstandings. To mitigate this issue, in this work, an alternative performance evaluation approach is chosen. More specifically, the SNR is calculated using a statistical approach which is based on

(17) $$SNR_{filt}=20\cdot log_{10}\left(\frac{2\cdot \Delta C_{in_{FS}}/4\cdot \sqrt{2}}{\sigma_{filt}}\right)=20\cdot log_{10}\left(\frac{\Delta C_{in_{FS}}/2\cdot \sqrt{2}}{\sigma_{filt}}\right)\left[\mathrm{dB}\right],$$

where a sinusoidal with an amplitude of $\pm \Delta C_{in_{FS}}$ (i.e., $2\cdot C_{in_{FS}}$ peak-to-peak) represents the signal. Consequently, $\Delta C_{in_{FS}}/2\cdot \sqrt{2}$ is the *RMS* value of such a signal. In Equation (17), $\sigma_{filt}$ yields the *RMS* noise, which equals the one-sigma standard deviation [16]. Thus, the Effective Number of Bits (ENOB) can be calculated via

(18) $$ENOB_{filt}=log_{2}\left(\frac{2\cdot \Delta C_{in_{FS}}}{\sqrt{12}\cdot \sigma_{filt}}\right)\left[\mathrm{bit}\right],$$

where $\Delta C_{in_{FS}}$ is the MEMS full scale sensitivity (Table 2). The factor 2 in the nominator is due to the fact that the CDC is a fully differential system which processes $\pm \Delta C_{in_{FS}}$. Special attention is again drawn to $\sigma_{filt}$, which represents the standard deviation of *many* (i.e., 1024) consecutive single (averaged) measurements. Ideally, for a constant sensor bridge, the CDC should always produce the same averaged digital output. Thus, the statistical variation of this digital output ($\sigma_{filt}$) gives information about the effective resolution of the CDC. Equation (18) calculates the ENOB of this CDC based on $\sigma_{filt}$.

**Figure 13:**
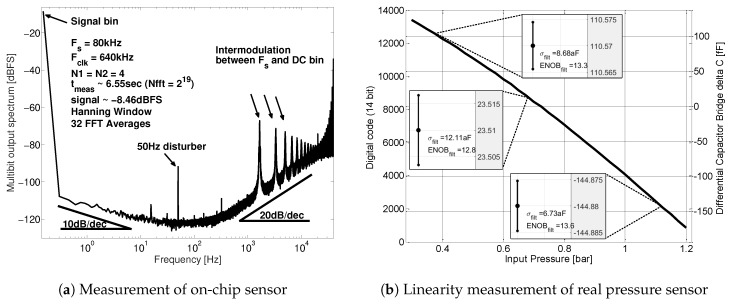
Long-term spectral approximation of on-chip dummy bridge measurement to reveal circuit characteristics such as noise-shaping, flicker-noise, tonal behavior and potential measurement disturbers (**a**); CDC system linearity plot (CDC + real pressure sensor): input pressure vs. digital reading and equivalent bridge capacitor difference, respectively. The pressure step size is 25 mbar and $\sigma_{filt}$ is after 1024 consecutive measurements (**b**).

Figure 13a shows a 32 times averaged spectral approximation of the 3-bit digital output $D_{OUT}$. This measurement was performed using an on-chip programmable capacitor bridge to evaluate the CDC performance. First-order noise-shaping and flicker-noise can be identified. The DC signal represents the differential bridge capacitance. Intermodulation tones between the DC signal and the clock are also present. This modulation effect is well known for first-order noise-shaping modulators. It does not affect the overall performance of the CDC as it only generates high frequency tones. The 50 Hz disturber can be associated with the external reference voltage $V_{REF}$.

### 7.1. Full Input Pressure Range Measurement

Next, a chip consisting of the CDC and a real pressure sensor MEMS (Figure 10b) was attached to a high-resolution pressure chamber. The on-chip offset calibration capacitors $C_{offset}$ are able to center the CDC input range around 0.75 bar with sufficient accuracy. Figure 13b shows the input pressure vs. the digital reading and the equivalent bridge capacitor difference, respectively. The input pressure step size of Figure 13b is 25 mbar within the range of 0.3–1.2 bar. The resolution is again calculated according to Equation (18). The measurement variation $\sigma_{filt}$ in Figure 13b is again based on 1024 subsequent measurements. A permanent resolution above 13 bit is observed over the full input pressure range.

Taking a closer look at both high and low input pressure edges in Figure 13b reveals that neither reaches the full scale (0–$2^{14}$ or $\Delta C_{in_{FS}}=\pm 193$ fF). This is due to the fact that the full scale calibration is bound to the resolution of the programmable $C_{DAC}$ array. A slightly lower DAC capacitor may exploit the sensor full scale even better.

Another important aspect of the CDC is linearity. Note that the measured sensor capacitance difference over the full scale in Figure 13b is not perfectly linear. Intrinsically, as soon as a MEMS is attached to the ASIC, any non-linearity of the sensor is measured too. Section 3 points out the sensor non-linearity based on a physical model of the sensor. Additionally, Infineon in-house measurements using a different ADC readout circuits paired to the same MEMS show similar curvature. It is concluded that Figure 13b shows the MEMS non-linearity due to varying MEMS stiffness rather than any CDC non-linearity. The potential effect of parasitic stray capacitance is not targeted within this work. Refer to [17,18] for further details on compensating non-linearity and parasitic parameters in resistive and capacitive sensor bridges.

### 7.2. Dead Zones

**Figure 14:**
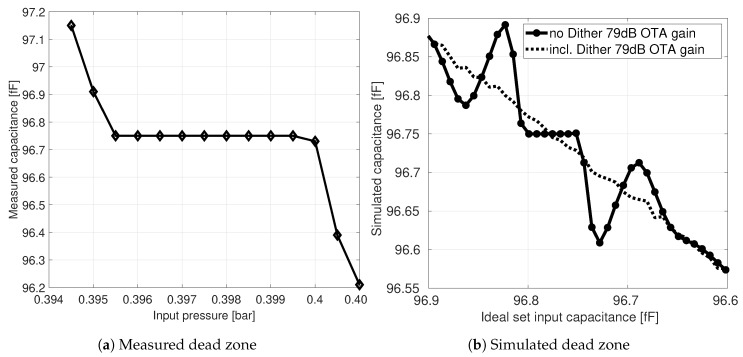
Dead zone example within measurement around 400 mbar of a real pressure sensor MEMS bridge (**a**); Simulated dead zone with and without added dithering (**b**).

It can be shown that the noise-shaping switched-capacitor CDC implementation in Section 5 is very similar to a conventional switched-capacitor single-bit first-order ΔΣ modulator with a counter. The main difference is that the input and feedback path contribution is split into two clock half phases. This is further verified by the appearance of *dead zones* in the measurements, as shown in Figure 14a. Several details on this phenomenon for first-order noise-shaping systems can be found in the literature [19]. Indeed, measurements show that dead zones exists around rational values of the input full scale. At those inputs, the final integrator output voltage after Phase II tends to be around zero due to the intended oscillation described in Section 5.2. Among other things, this mainly challenges the comparator accuracy.

It is interesting to point out that the appearance of dead zones can also be simulated. Figure 14b shows the same dead zone at $\Delta C_{FS}/4$ when no dithering is present. Due to the higher input increment resolution, further non-linear effects are apparent. This simulation was implemented via MATLAB^®^ code where the delta voltage steps (Equations (12) and (13)) are pre-calculated and virtually stepped through. The equations also involve an influence on the OTA DC gain. Likewise, the literature suggests that the width of a dead zone is inverse proportional to the DC gain. However, simulations based on the simplified model do not show such a dependency. The width of the dead zone stayed rather constant. A possible reason for this is that, although each voltage step is modeled correctly, it does not represent a more precise leaky integrator model such as in SIMULINK^®^. Adding dithering in front of the virtual comparator indeed reduces the effect of dead zones, as indicated in Figure 14b.

### 7.3. Performance Scaling Potential

In a previous study, performance scaling capability was predicted [20]. It was shown that the maximum Signal-to-Noise Ratio (SNR) of a first-order noise-shaping system is estimated [19]

(19) $$SNR=6.02\cdot B_{bits}+30\cdot log_{10}\left(\frac{f_{m}}{2\cdot f_{BW}}\right)-3.41,$$

where $B_{bits}$ is the number of bits used in the quantizer. Equation (19) actually assumes sinusoidal input signals and a certain signal bandwidth $f_{BW}$. As discussed in Section 7, this is controversial for DC signal. However, when selecting $f_{BW}$ properly, Equation (19) also offers a good approximation for DC signals. More specifically, $f_{BW}=1/T_{m_{total}}$ is selected to define the signal bandwidth of a DC signal based on the measurement time in Equation (14). Another analogy in Equation (19) to the proposed Dual-Slope approach is found by defining $B_{bits}=log_{2}\left(M\right)+1$, with *M* being the number of clock cycles during Phase II. The additional bit is due to the intrinsic sign bit of the differential output. Equation (19) can then be mapped to the Effective Number of Bits (ENOB) via

(20) $$ENOB=\frac{SNR-1.76}{6.02}.$$

**Figure 15:**
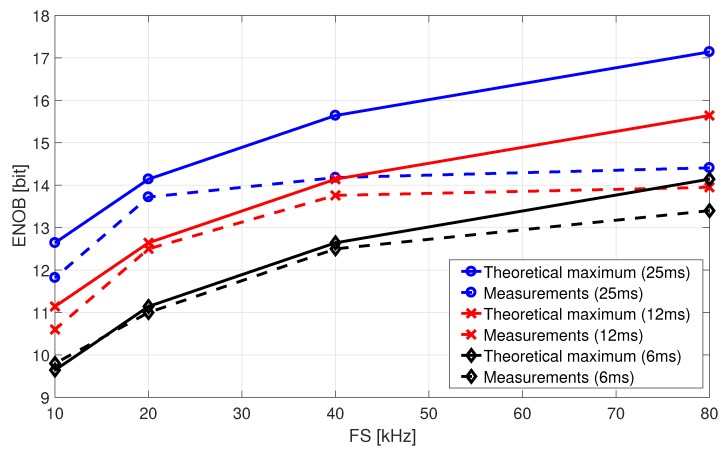
Performance scaling potential of measured CDC using 1024 consecutive measurements applying a on-chip capacitor dummy bridge at ≈−2 dBFS.

Equation (19) predicts performance scaling potential by simply changing the clock frequency and/or the measurement time, which is common in averaging converter approaches. In general the CDC measurements are able to proof this scalability on silicon. Note that those measurements are performed on a on-chip dummy MEMS sensor bridge to exclude additional physical sensor effects. Figure 15 compares the theoretical maximum and measured performance for different measurement times over different sampling frequencies. For each data point, Equation (18) is applied using 1024 subsequent measurements for deriving $\sigma_{filt}$. The higher the sampling frequency, the more samples per time are captured and the better the performance. Clearly, the maximum performance of a real implementation is limited by noise. Note, however, that the average value of pure white noise is zero and should not affect the CDC performance scaling. Measurements show that the main limitation of highly averaged scenarios in Figure 15 is due to flicker-noise (1/f). This is confirmed by the long-term measurement in Figure 13a. While simplified circuit-level Periodic-Noise (PNoise) simulations indeed show a reduction of the OTA flicker-noise contribution, the residual 1/f noise still dominates for DC signals.

### 7.4. Comparison to State of The Art

To compare the efficiency among different implementations, it is common to use the following Figure of Merit (FoM). In terms of power vs. performance, this paper applies

(21) $$FoM=\frac{Power\cdot T_{m_{total}}}{2^{ENOB_{filt}}}\left[\frac{\mathrm{pJ}}{\mathrm{step}}\right].$$

Measuring 3.2 ms at a multi-bit rate $f_{m}$ = 80 kHz does K = 256 conversions to give one averaged measurement result. This single measurement has been repeated for 1024 times to derive the variation of the measurement results ($\sigma_{filt}$) to apply Equation (18). The CDC consumes 35 μA from a 1.5 V power supply. This current includes the analog and digital blocks without the external reference. Equation (18) gives 13 bit with the on-chip bridge at a fixed bridge signal. This resolution represents the effect of noise while measuring a constant bridge. According to Equation (21), this yields a FoM of 20.6 pJ/step. Table 3 shows a comparison of selected state of the art CDCs and this work. A large variation of the applied FoM can be observed. Interestingly, this variation persists even among the same type of converter. An exception to this can be observed for the hybrid solutions, which indicates the future trend in CDC development. Note that the specified capacitor range in Table 3 refers to the maximum expected sensitivity range of our sensor. The absolute size of the sensor capacitors play a secondary role in terms of load at the virtual OTA ground and total sensor current consumption.

**Figure 16:**
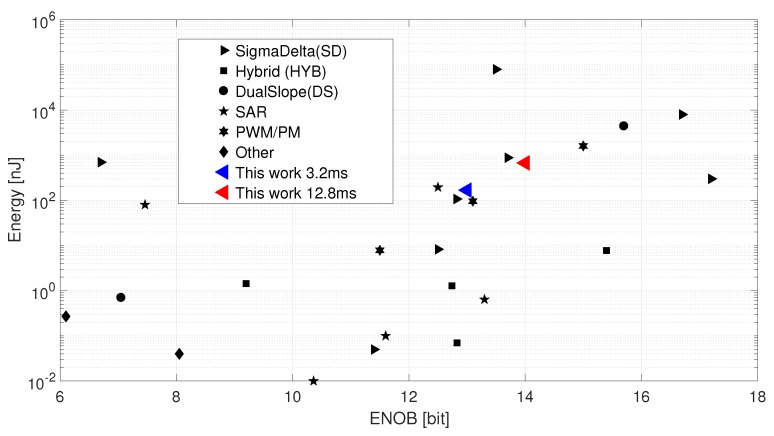
State of the art energy vs. resolution comparison.

Another visual state-of-the-art comparison is shown in Figure 16. It plots the achieved resolution versus the energy being used of Table 3. A clear trend of higher energy consumption for higher resolution is observed. The FoM of the proposed converter is in the range of other CDCs. Higher energy consumption by extending the measurement time also improves the resolution, as discussed in Section 7.3. This moves the FoM indicator in Figure 16 along a virtual line from the lower left to the upper right achieving 14 bit resolution. The closely situated ΔΣ approach in Figure 16 also consists of a first-order system achieving similar resolution. Again, the superior performance of hybrid solutions is observed.

## 8. Conclusions and Dual-Slope CDC Outlook

This article reports details about a switched-capacitor noise-shaping Dual-Slope CDC. The main focus is put on the measurement setup and results. Special auxiliary hardware was used to measure a real pressure sensor in a controlled ambient air pressure environment. The silicon proved CDC showed performance scaling capability thanks to the averaging concept. Apparent dead zones relate to first-order systems documented in the literature. Furthermore, this article discusses physical MEMS basics and a SPICE model approach of an ambient air pressure sensor. It shows how basic physical equations lead to a MEMS sensor model that can be used in a circuit design simulator. Naturally, further improvements can be found to increase efficiency of the Dual-Slope CDC. For example, it can be shown that N=1 (i.e., Phase I of the Dual-Slope approach only lasts one clock cycle) yields the best sensor current consumption, since the differential bridge is only pre- and discharged once per conversion cycle. Additionally, it minimizes the conversion time for a given multi-bit scenario according to Equation (11). Recognizing the power of hybrid solutions in Section 7.4, further enhancements can be implemented. In [36], the single-bit conversion in Phase II is replaced by a SAR concept. This further reduces the conversion time while using a binary weighted multi-bit approach towards a hybrid solution.

## Figures and Tables

**Table 1:** Given pressure sensor MEMS sensitivity estimations.

	[unit]	MIN	NOM	MAX
Sensor capacitor $C_{sen}$ absolute size	[pF]	4.68	5.71	6.74
Reference capacitor $C_{ref}$ absolute size	[pF]	4.58	5.45	6.32
$C_{sen}$ full scale variation (0.3–1.2 bar)	[fF]	242.1	526.3	810.5
$C_{ref}$ full scale variation (0.3–1.2 bar)	[fF]	6.9	17.3	27.8
Linearized $C_{sen}$ sensitivity (over 1 bar)	[fF/bar]	269.0	584.8	900.6
Linearized $C_{ref}$ sensitivity (over 1 bar)	[fF/bar]	7.7	19.3	30.9
Linearized $\Delta C=C_{sen}-C_{ref}$ sensitivity	[fF/bar]	261.3	565.5	869.8
**Effective sensor full scale** $\Delta C_{\mathrm{FS}}$ **(0.3–1.2 bar)**	[fF]	**235.2**	**508.9**	**782.8**
**Centered equivalent sensor full scale** $\pm \Delta C_{\mathrm{FS}}$	[fF]	±117.6	±254.5	±391.4

**Table 2:** CDC circuit design parameters for real pressure sensor MEMS connected.

Parameter	Value	Parameter	Value
OTA DC gain (*A*)	79 dB	OTA $GBW_{LG}$	2.3 MHz
Supply voltage	1.5 V	OTA supply current	28 μA
$\Delta C_{in_{FS}}$	±148.5 fF	$C_{sen}\approx C_{ref}$	5.7 pF
$C_{DAC}$	387 fF	$C_{F}$	4.52 pF
$C_{AZ}$	2 pF	*N* = *M*	4
$V_{REF}$	1.5 V	$V_{CM}$	750 mV

**Table 3:** Comparison with State-of-the-art CDCs peak FoM (Equation (21)).

Ref.	Type	Measurement	Power	Capacitor	ENOB	FoM
		Time [sec]	[Watt]	Range [pF]	[bit]	[pJ/step]
[6]	ΔΣ	20 μ	15 m	10	17.2	2
[21]	ΔΣ	13.3 m	6 m	0.16	13.5	6904
[22]	ΔΣ	0.8 m	10 μ	0.5	12.5	1.4
[23]	ΔΣ	10.2 m	10.5 μ	2	12.8	14.9
[24]	ΔΣ	100 m	7 μ	0.4	6.7	6725
[25]	ΔΣ	1 m	882 μ	N.A.	13.7	66.3
[26]	ΔΣ	10.5 m	760 μ	16	16.7	75
[27]	HYB	0.23 m	34 μ	24	15.4	0.2
[28]	HYB	1 μ	1.44 m	1	9.2	2.4
[29]	HYB	0.81 m	1.59 μ	3.6	12.74	0.188
[14]	DS	20 m	220 μ	1	15.7	82.7
[7]	DS	6.4 m	110 n	11.3	7	5.3
[2]	SAR	4 m	160 n	61	13.3	0.1
[30]	SAR	100 m	800 n	18.5	7.46	455
[31]	SAR	1 μ	7.5 μ	5	10.36	0.0055
[32]	SAR	0.65 m	300 μ	15	12.5	33.7
[33]	SAR	16 μ	6.44 μ	12.66	11.6	0.0332
[4]	PWM	80 μ	98 μ	22	11.5	2.7
[3]	PM	7.6 m	211 μ	6.8	15	49
[34]	PM	6.8 m	14 μ	2.22	13.1	10.9
[35]	Dig	1 m	270 n	0.3	6.1	3.9
[5]	DC	19 μ	1.84 μ	11.3	8	0.1
**This** **work**	DS	3.2 m	52.8 μ	0.9	13	20.6

## Abbreviations

The following abbreviations are used in this manuscript:

SPICE	Simulation-Program-with-Integrated-Circuit-Emphasis
TPMS	Tire-Pressure-Monitoring-System
RLC	Resistive-Inductive-Capacitive
DOF	Degree-Of-Freedom
FS	Full-Scale
ASIC	Application-Specific-Integrated-Circuit
CDC	Capacitance-to-Digital Converter
LDO	Low-DropOut generator
ADC	Analog-to-Digital Converter
MEMS	Micro-Electro-Mechanical-System
SC	Switched-Capacitor
OTA	Operational-Transconductance-Amplifier
ΔΣ	Delta Sigma modulator
HYB	Hybrid converter
DS	Dual-Slope converter
SAR	Successive-Approximation-Register
PWM	Pulse-Width-Modulation
PM	Period-Modulation
DC	Digital-Converter
